# Industrial Food Systems as Environmental Drivers of Women’s Health and Childhood Obesity: A Cross-National Ecological Study

**DOI:** 10.3390/nu18091435

**Published:** 2026-04-30

**Authors:** Myriam Angélica Castiblanco-Amaya, Laia Selva-Pareja, Jonh Jairo Méndez-Arteaga, Elena Paraíso-Pueyo, Rosa Mar Alzuria-Alós, Anna Espart

**Affiliations:** 1 Department of Public Health, University of Tolima, Ibagué 730001, Colombia; mcastiblanco@ut.edu.co; 2 Research Group on Health Self-Care (GIACSUT), University of Tolima, Ibagué 730001, Colombia; 3 Department of Nursing and Physiotherapy, University of Lleida, 25198 Lleida, Spain; elena.paraiso@udl.cat (E.P.-P.); rosamar.alzuria@udl.cat (R.M.A.-A.); anna.espart@udl.cat (A.E.); 4 Health Education, Nursing, Sustainability and Innovation Research Group (GREISI), Lleida Institute for Biomedical Research Dr. Pifarré Foundation (IRBLleida), 25198 Lleida, Spain; 5 Health Education, Nursing, Sustainability and Innovation Research Group (GREISI), University of Lleida, 25198 Lleida, Spain; 6 Development of Healthy and Sustainable Organizations and Territories (DOTSS), University of Lleida, 25001 Lleida, Spain; 7 Science Faculty, University of Tolima, Ibagué 730001, Colombia; jmendez@ut.edu.co; 8 Natural Products Research Group (GIPRONUT), University of Tolima, Ibagué 730001, Colombia

**Keywords:** childhood obesity, women’s health, female obesity, industrial food systems, food environments, sugar availability, intergenerational obesity, environmental health, cross-national study

## Abstract

**Background**: Childhood obesity is a growing global public health concern influenced by early-life conditions and broader environmental factors. Although industrial food systems shape dietary patterns, cross-national ecological evidence on how these environments influence childhood obesity through population-level pathways related to women’s health remains limited. **Objective**: This study aims to examine whether industrial food systems influence childhood obesity through population-level pathways involving women’s health, and to assess whether female obesity mediates the association between national sugar availability and childhood obesity prevalence. **Methods**: A cross-national ecological study was conducted using publicly available data from 46 countries (2015–2020). Childhood obesity (ages 5–19 years) and female obesity (≥18 years) were obtained from the WHO Global Health Observatory. Food system indicators were derived from FAOSTAT, and socioeconomic variables from the World Bank. Pearson correlations, multivariable regression models, and mediation analysis with bootstrapping (5000 resamples) were performed. **Results**: Female obesity was strongly associated with childhood obesity prevalence (r = 0.71, *p* < 0.001), explaining approximately 50% of its variance. Sugar availability was positively associated with both female obesity and childhood obesity (r = 0.49 for both associations; *p* < 0.001). In multivariable models, female obesity remained the only significant predictor (β = 0.61, *p* < 0.001), with the model explaining 59% of the variance (R^2^ = 0.59; adjusted R^2^ = 0.49). Mediation analysis showed a significant indirect effect of sugar availability on childhood obesity through female obesity (B = 0.013, *p* = 0.003), with no significant direct effect. **Conclusions**: These findings support a population-level framework in which industrial food systems may influence intergenerational obesity through pathways involving women’s health. Female obesity may act as an integrative marker linking food environments to childhood obesity risk.

## 1. Introduction

Childhood obesity has emerged as a major global public health concern, with prevalence increasing substantially over recent decades across both high- and middle-income countries [1,2]. Recent estimates indicate that childhood overweight and obesity continue to rise across both high-income and middle-income countries, while low-income settings increasingly face a double burden of undernutrition and obesity during nutritional transition [1,2]. Excess weight during childhood is associated with a wide range of adverse health outcomes, including cardiometabolic disorders, type 2 diabetes, and increased risk of obesity in adulthood [3,4,5,6]. Importantly, early-life conditions play a critical role in shaping obesity risk, highlighting the importance of identifying determinants operating during pregnancy and early childhood [7,8]. Determinants of childhood obesity are multifactorial and include early-life exposures, family dietary environments, urbanization, socioeconomic inequalities, and broader food system conditions [9,10,11].

Maternal health and nutritional status during pregnancy represent key early-life influences on child growth and metabolic development. Maternal obesity has consistently been associated with higher risk of childhood obesity through a combination of biological, behavioral, and environmental pathways [12,13]. Maternal diet during pregnancy may influence fetal programming and postnatal growth trajectories, while shared family environments and dietary patterns may further reinforce intergenerational patterns of obesity [14,15,16]. Childhood obesity is additionally influenced by broader contextual factors such as urbanization, socioeconomic inequalities, healthcare access, and lifestyle environments, which should be considered when interpreting ecological associations [10].

Beyond individual dietary choices, nutritional exposures among women are shaped by broader food system environments that influence the availability and consumption of specific food products at the population level [17,18]. Industrial food systems are also characterized by food system transformation processes, including the globalization of food supply chains, widespread availability of ultra-processed products, increasing market concentration, and policy environments that shape national food landscapes [17,18,19,20]. These systems often promote high availability of refined sugars, fats, and energy-dense foods, which may influence population-level dietary patterns and obesity prevalence [21,22]. At the population level, obesity among adult women may serve as an integrative marker of dietary environments affecting women of reproductive age, providing a potential link between food systems and intergenerational health outcomes [23]. As women of reproductive age are directly exposed to these food environments before and during pregnancy, population-level female obesity may capture cumulative nutritional and metabolic conditions relevant to intergenerational obesity risk [23,24,25].

Among food system indicators, national sugar availability may be particularly relevant as a marker of obesogenic dietary environments. Higher per capita availability of sugar and sweeteners often reflects greater access to sugar-rich processed foods and beverages, which have been consistently associated with excess energy intake, weight gain, and obesity risk [21,22,26,27]. At the population level, such environments may influence dietary exposures among both women of reproductive age and children, making sugar availability a useful proxy for examining intergenerational obesity pathways [23].

Despite increasing recognition of the role of food systems in shaping population diets, relatively few studies have examined how national food system environments may influence childhood obesity through pathways operating at the level of women’s health. Most previous studies have focused on individual-level behavioral or maternal risk factors. Cross-national evidence simultaneously examining food system indicators, female obesity, and childhood obesity remains limited, particularly using mediation approaches that may help clarify potential population-level pathways [10,12,13,19].

The aim of this study was to examine associations between industrial food system indicators, female obesity, and childhood obesity across countries. We further explored whether female obesity mediates the association between national sugar availability and childhood obesity prevalence while accounting for relevant national contextual factors. By integrating food system exposures, women’s health, and childhood obesity within a cross-national ecological framework, this study provides novel population-level evidence on potential intergenerational pathways linking food environments to obesity risk and highlights the public health relevance of addressing obesity across generations.

## 2. Materials and Methods

### 2.1. Study Design

This study employed a cross-national ecological design to examine associations between indicators of industrial food systems, female obesity, and childhood obesity across countries. Publicly available international datasets were used to construct a country-level analytical dataset covering the period 2015–2020. The study aimed to explore environmental food system contexts potentially influencing women’s health and early-life obesity patterns at the population level.

### 2.2. Data Sources

Data were obtained from several publicly available international databases that use standardized data collection, harmonization, and quality control procedures. Childhood obesity prevalence among children and adolescents aged 5–19 years was obtained from the World Health Organization (WHO) Global Health Observatory, which compiles nationally reported and standardized international health indicators. The indicator corresponds to the prevalence of obesity defined as BMI-for-age greater than +2 standard deviations above the WHO growth reference.

Female obesity prevalence was also obtained from the WHO Global Health Observatory, defined as the prevalence of obesity (BMI ≥ 30 kg/m^2^) among women aged 18 years and older.

Indicators of national food system availability were obtained from FAOSTAT Food Balance Sheets, which provide harmonized national estimates of food supply availability, including per capita daily availability of sugar and sweeteners and vegetable oils, expressed as kilocalories per capita per day.

Agricultural pesticide use intensity (kg per hectare of cropland) was also obtained from FAOSTAT.

Country-level contextual indicators were obtained from the World Bank Open Data platform, which provides internationally comparable socioeconomic indicators, including gross domestic product (GDP) per capita, percentage of population living in urban areas, and female educational attainment.

Data corresponding to the period 2015–2020 were compiled to represent recent national conditions. When multiple years were available within this time frame, values were averaged across available years within the study period to represent country-level indicators.

### 2.3. Study Sample

A total of 46 countries were included in the descriptive analyses. Countries were eligible for inclusion if data were available for childhood obesity and at least one of the primary food system exposure variables during the study period. Countries with missing data for the outcome variable were excluded. The list of included countries is provided in Supplementary Table S1. Missing data across covariates were primarily due to incomplete reporting in the original international databases and may not have occurred at random.

The study included countries spanning low-, lower-middle-, upper-middle-, and high-income categories according to World Bank classifications, reflecting diverse socioeconomic and food system contexts.

Regression analyses were conducted on countries with complete data for the variables included in each model. Consequently, the effective sample size varied across statistical analyses.

### 2.4. Variables

The primary outcome variable was childhood obesity prevalence among children and adolescents aged 5–19 years.

The main exposure variables representing national food system contexts included:

- Per capita availability of sugar and sweeteners (kcal per capita per day);
- Per capita availability of vegetable oils (kcal per capita per day);
- Agricultural pesticide use intensity (kg per hectare).

Female obesity prevalence among women aged ≥18 years was included as a key population-level indicator of women’s health, reflecting cumulative nutritional and metabolic conditions relevant to women of reproductive age. At the ecological level, this variable was considered a plausible intermediate marker linking national food environments with childhood obesity risk through intergenerational biological and shared environmental pathways.

Additional country-level contextual variables included urbanization (percentage of population living in urban areas), GDP per capita, and female educational attainment.

Because pesticide use showed a highly skewed distribution across countries, a logarithmic transformation was applied prior to regression analyses.

### 2.5. Statistical Analysis

Descriptive statistics were calculated for all variables, including means, standard deviations, and ranges.

Pearson correlation analyses were conducted to examine the strength and direction of bivariate associations between childhood obesity and country-level variables.

Linear regression models were used to estimate associations between food system indicators and childhood obesity prevalence. Both univariate and multivariable regression models were estimated. Multivariable models included food system indicators and contextual variables to assess whether associations persisted after adjustment.

To explore potential mechanisms linking food system exposures with childhood obesity, mediation analysis was conducted using a regression-based approach to assess whether female obesity mediated the association between national sugar availability and childhood obesity prevalence. Indirect, direct, and total effects were estimated, and confidence intervals were obtained using bootstrapping with 5000 resamples.

Variables were analyzed in their original units unless otherwise specified. Standardized coefficients (β) are presented in regression models to facilitate comparison of effect sizes. Potential outliers and influential observations were examined using scatterplots, standardized residuals, leverage statistics, and Cook’s distance. No observations were excluded solely on the basis of these diagnostics.

All statistical analyses were conducted using Jamovi (v2.7.16) statistical software. Statistical significance was defined as *p* < 0.05.

## 3. Results

### 3.1. Descriptive Characteristics of the Study Sample

A total of 46 countries were included in the descriptive analyses, representing diverse economic contexts spanning low-, lower-middle-, upper-middle-, and high-income categories according to World Bank classifications. Across countries, the mean prevalence of childhood obesity among children and adolescents aged 5–19 years was 9.4% (SD = 4.9), ranging from 1.3% to 24.9%.

Female obesity prevalence among women aged ≥18 years averaged 22.2% (SD = 12.0). National sugar availability averaged 299 kcal per capita per day, while vegetable oil availability averaged 44.8 kcal per capita per day. Agricultural pesticide use intensity varied substantially across countries.

Detailed descriptive statistics for all study variables are presented in Table 1.

### 3.2. Association Between Female Obesity and Childhood Obesity

Across countries, female obesity prevalence showed a strong positive association with childhood obesity prevalence. Pearson correlation analysis indicated a strong correlation between female obesity and childhood obesity (r = 0.71, *p* < 0.001).

In univariate regression analyses, female obesity was the strongest predictor of childhood obesity prevalence across countries (β = 0.292, *p* < 0.001), explaining approximately 50% of the variance in childhood obesity prevalence (R^2^ = 0.50).

**Figure 1:**
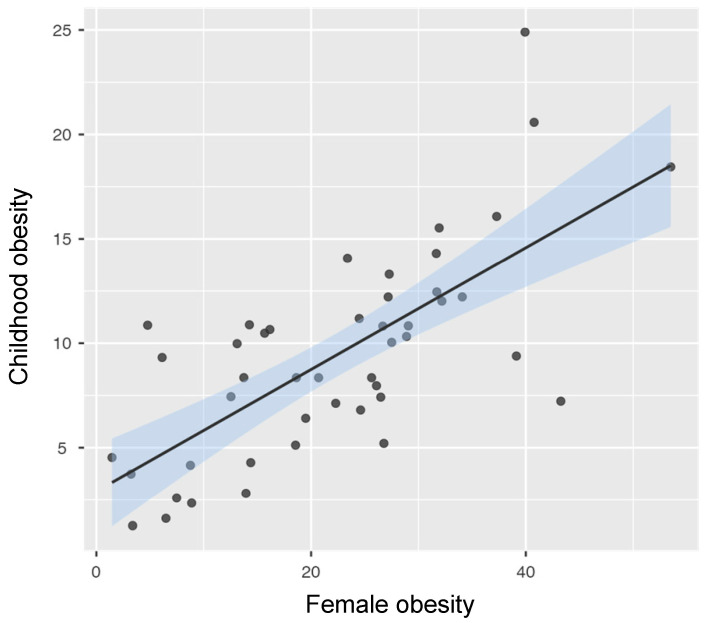
Scatterplot showing the association between female obesity prevalence and childhood obesity prevalence. The solid line represents the fitted linear regression, and the shaded area indicates the 95% confidence interval.

The relationship between female obesity and childhood obesity across countries is illustrated in Figure 1.

### 3.3. Food System Indicators and Obesity Patterns

Indicators of national food systems were also associated with obesity patterns across countries. Sugar availability showed a moderate positive correlation with childhood obesity prevalence (r = 0.49, *p* < 0.001) and with female obesity prevalence (r = 0.49, *p* < 0.001).

Vegetable oil availability showed a weaker but significant correlation with childhood obesity (r = 0.31, *p* = 0.040). After logarithmic transformation, pesticide use was also positively correlated with childhood obesity in bivariate analyses (r = 0.38, *p* < 0.01). In contrast, GDP per capita was not significantly associated with childhood obesity prevalence. Female educational attainment was also not significantly associated with childhood obesity in bivariate analyses. However, the association between pesticide use and childhood obesity did not remain statistically significant in multivariable models.

The full correlation matrix between childhood obesity and country-level variables is presented in Table 2.

**Figure 2:**
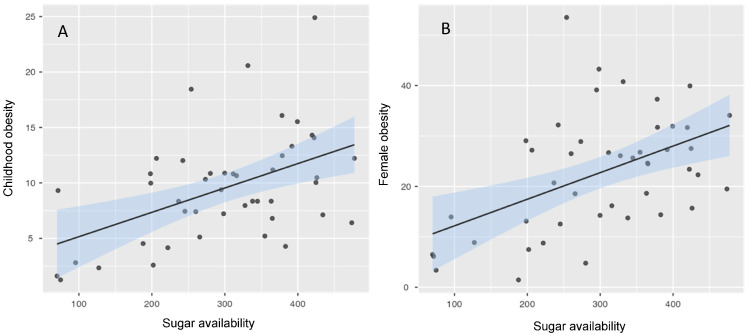
Scatterplot showing the association between sugar availability and obesity prevalence. (**A**): childhood obesity; (**B**): adult female obesity. The solid line represents the fitted linear regression, and the shaded area indicates the 95% confidence interval.

Scatterplots illustrating the relationships between sugar availability, adult female obesity, and childhood obesity are presented in Figure 2.

### 3.4. Multivariable Regression Models

Multivariable regression models were constructed to examine whether associations between food system indicators and childhood obesity persisted after adjusting for contextual factors and female obesity.

In models including food system indicators alone, sugar availability remained positively associated with childhood obesity prevalence. However, after female obesity was included in the model, female obesity emerged as the strongest independent predictor of childhood obesity (β = 0.61, *p* < 0.001). The full model explained approximately 59% of the variance in childhood obesity prevalence across countries (R^2^ = 0.59; adjusted R^2^ = 0.49; N = 36). None of the other predictors remained statistically significant in the fully adjusted model. Results of the multivariable regression analyses are presented in Table 3.

### 3.5. Mediation Analysis

To explore potential mechanisms linking food system environments to childhood obesity, mediation analysis was conducted to assess whether female obesity mediated the association between national sugar availability and childhood obesity prevalence.

The total effect of sugar availability on childhood obesity was statistically significant (B = 0.022, *p* < 0.001). The indirect effect through female obesity was also significant (B = 0.013, *p* = 0.003), while the direct effect was not statistically significant after accounting for female obesity (B = 0.009, *p* = 0.110).

These findings suggest that the association between sugar availability and childhood obesity may be largely mediated by female obesity at the population level. Results of the mediation analysis are presented in Table 4.

## 4. Discussion

### 4.1. Principal Findings

This cross-national ecological study examined associations between indicators of industrial food systems, female obesity, and childhood obesity across countries. Several important findings emerged. Taken together, these findings support a pathway in which industrial food systems influence childhood obesity indirectly through their impact on women’s health at the population level.

First, female obesity showed a strong association with childhood obesity prevalence across countries, explaining a substantial proportion of the variance in childhood obesity rates. Second, national sugar availability was positively associated with both female obesity and childhood obesity in univariate analyses. Third, mediation analysis suggested that female obesity significantly mediated the relationship between national sugar availability and childhood obesity prevalence.

Together, these findings suggest that food system environments may shape childhood obesity partly through pathways operating at the level of women’s health.

### 4.2. Food Systems, Diet, and Women’s Health Pathways

A key implication of these findings relates to the role of daily dietary environments shaped by national food systems. Indicators such as national sugar availability reflect broader food supply conditions that influence population-level dietary intake patterns [22]. In many countries, industrial food systems are characterized by high availability of refined sugars and energy-dense products, which contribute to increased consumption of added sugars and ultra-processed foods [20,22,28]. These food supply conditions are likely reflected in daily dietary intake, contributing to higher energy consumption and poorer diet quality at the population level [29].

Although this study did not directly assess maternal diet, these population-level dietary environments are highly relevant for women of reproductive age. High availability of refined sugars and energy-dense foods may increase the likelihood of excessive caloric intake and weight gain among adult women [26,27]. In this context, female obesity may serve as a proxy for nutritional and metabolic conditions relevant to maternal health, representing a potential pathway linking population-level dietary environments to childhood obesity risk [23,30].

These findings are consistent with previous research suggesting that maternal nutritional status and diet during pregnancy play a critical role in shaping early-life metabolic programming and subsequent obesity risk in offspring [14,24,25]. Shared family dietary environments after birth may further reinforce these intergenerational patterns of obesity [11,31].

Together, these findings support the relevance of women’s health as a key pathway through which obesogenic food environments may influence childhood obesity risk.

### 4.3. Environmental Food Contexts and Global Obesity Patterns

The results of this study also contribute to the growing literature examining the role of food systems and environmental dietary contexts in shaping population health [19]. National food supply indicators such as sugar availability provide insight into the structure of food environments that influence dietary patterns across populations.

Countries with higher availability of refined sugars and industrial food products may experience dietary shifts toward higher consumption of energy-dense foods and beverages. These dietary transitions are widely recognized as part of the global nutrition transition and have been linked to rising obesity prevalence in many regions [31].

By examining associations across countries with diverse economic and food system contexts, the present study highlights how structural characteristics of food systems may shape dietary exposures relevant to women’s health and early-life obesity patterns.

These results reinforce the importance of considering structural food environments when addressing rising obesity prevalence across countries.

### 4.4. Interpretation of Non-Significant Environmental Indicators

In contrast to sugar availability, agricultural pesticide use was not significantly associated with childhood obesity in multivariable models. Although a significant correlation was observed in bivariate analyses after log transformation, this association did not persist after adjustment for other variables. This suggests that pesticide use may reflect broader characteristics of industrial food systems rather than an independent pathway influencing obesity risk. Pesticide use may therefore act as a proxy for agricultural intensification within industrial food systems [32,33].

Moreover, dietary patterns and caloric intake likely represent more direct pathways linking food systems to obesity outcomes than agricultural production indicators alone [34]. These findings suggest that food supply characteristics influencing daily dietary intake may play a more prominent role in shaping population obesity patterns.

Similarly, vegetable oil availability showed a modest association with childhood obesity in bivariate analyses, but this relationship did not persist in multivariable models. This suggests that vegetable oil availability may reflect broader dietary patterns within industrial food systems, but does not independently explain variations in childhood obesity across countries.

Overall, these findings suggest that downstream dietary exposures may be more strongly linked to childhood obesity than upstream agricultural indicators alone.

### 4.5. Strengths and Limitations

This study has several strengths. By combining data from multiple international sources, the analysis included countries with diverse economic and food system contexts. The use of multiple indicators allowed examination of relationships between food systems, women’s health, and childhood obesity at the population level.

However, several limitations should also be considered. First, the ecological design does not allow inference about individual-level causal relationships, and associations observed at the country level may not reflect individual-level mechanisms (ecological fallacy) [35]. Residual confounding from unmeasured country-level factors cannot be excluded. Second, national food supply indicators may not fully capture individual dietary intake patterns. Third, female obesity was used as a proxy for maternal health, and not all women included in this indicator are mothers, limiting direct interpretation of maternal pathways. Finally, differences in data availability across countries resulted in varying sample sizes across statistical models.

Therefore, these findings should be interpreted as hypothesis-generating rather than causal; however, cross-national ecological analyses can still provide valuable insights into structural and environmental factors shaping population health patterns.

### 4.6. Implications for Public Health

The findings of this study suggest that efforts to address childhood obesity may benefit from considering women’s health and dietary environments simultaneously. Policies targeting population dietary patterns—such as reducing the availability and consumption of added sugars and ultra-processed foods—may contribute to improvements in both women’s and child health outcomes [36,37].

In this context, female obesity may represent not only an intermediate marker of obesogenic environments, but also a potentially modifiable factor within the pathway linking food systems to childhood obesity. Improvements in women’s nutritional status and metabolic health before and during the reproductive years may help reduce childhood obesity risk, even in settings where adverse food environments persist [23,24,25]. However, interventions focused solely on individual behavior are unlikely to be sufficient without broader upstream changes in food systems that improve the availability, affordability, and accessibility of healthier foods [19,36,37].

Recognizing the influence of broader food system environments on daily dietary intake may therefore be important for designing effective strategies to prevent obesity across generations.

A combined focus on women’s health and structural food system reform may therefore offer a more effective strategy for long-term obesity prevention.

## 5. Conclusions

This cross-national ecological analysis suggests that industrial food system environments characterized by high sugar availability may influence childhood obesity partly through their association with female obesity. The findings highlight the potential role of daily dietary environments in shaping women’s health and intergenerational obesity patterns.

By linking food system indicators with female and childhood obesity across countries, this study underscores the importance of considering pathways related to women’s health when examining environmental influences on childhood obesity. Policies and interventions aimed at improving population dietary patterns—particularly reducing excessive sugar consumption—may contribute to preventing obesity across generations.

Further research is needed to better understand how food system environments influence maternal diet during pregnancy and how early-life nutritional exposures shape childhood obesity risk.

## Figures and Tables

**Table 1:** Descriptive statistics of study variables.

Variable	N	Mean (±SD)	Min; Max
Childhood obesity (%)	46	9.4 (±4.92)	1.26; 24.9
Female obesity (%)	46	22.2 (±12.0)	1.46; 53.5
Sugar availability (kcal/capita/day)	45	299 (±108)	70.0; 478
Vegetable oils * (kcal/capita/day)	45	44.8 (±17.1)	12.1; 94.4
Pesticides use (kg/ha)	32	4.5 (±4.37)	0.233; 21.4
Log Pesticides	32	0.978 (±1.16)	−1.46; 3.06
Urbanization (%)	46	68.7 (±18.2)	20.5; 95.3
GDP capita (USD)	46	29,487 (±20,932)	2255; 90,225
Female education ** (%)	36	89.5 (±14.5)	26.1; 104 ^a^

GDP: gross domestic product. * Vegetable oils primarily reflect refined oils commonly used in industrial food production. ** Lower secondary completion rate; ^a^: may exceed 100 due to data harmonization. Sample size varied due to missing data.

**Table 2:** Pearson correlation matrix of country-level variables.

Variable	Childhood Obesity	Female Obesity	Sugar Availability	Vegetable Oils	Log Pesticide	Urbanization	GDP	Female Education
Childhood obesity	—							
Female obesity	0.710 ***	—						
Sugar availability	0.485 ***	0.485 ***	—					
Vegetable oils	0.307 *	0.345 *	0.429 **	—				
Log Pesticide	0.377 **	0.213	0.477 ***	0.315	—			
Urbanization	0.367 *	0.328 *	0.555 ***	0.478 ***	−0.072	—		
GDP	0.181	0.100	0.346 *	0.526 ***	0.019	0.555 ***	—	
Female education	0.243	0.095	0.360 *	0.287	−0.036	0.513 **	0.493 **	—

* *p* < 0.05; ** *p* < 0.01; *** *p* < 0.001.

**Table 3:** Multivariable regression models examining associations between food system indicators, female obesity, and childhood obesity.

Predictor	B	SE	β	95% CI	*p*-Value
Sugar availability	0.002	0.009	0.046	−0.015, 0.020	0.779
Vegetable oils	−0.030	0.046	−0.099	−0.125, 0.064	0.518
Log Pesticide	0.913	0.598	0.205	−0.312, 2.138	0.138
Urbanization	0.026	0.051	0.087	−0.079, 0.131	0.613
GDP	0.00005	0.00004	0.182	−0.00004, 0.00014	0.276
Female education	0.002	0.054	0.005	−0.110, 0.113	0.975
Female obesity	0.261	0.058	0.614	0.142, 0.380	<0.001

B: unstandardized regression coefficient; SE: standard error; β: standardized regression coefficient; CI: confidence interval. Model R^2^ = 0.59; Adjusted R^2^ = 0.49; N = 36.

**Table 4:** Mediation model examining the role of female obesity in the association between sugar availability and childhood obesity.

Effect	B	SE	95% CI	*p*-Value
Total effect	0.022	0.006	0.010, 0.035	<0.001
Direct effect	0.009	0.005	−0.001, 0.020	0.110
Indirect effect	0.013	0.004	0.005, 0.023	0.003

B: unstandardized coefficient; SE: standard error; CI: confidence interval. The indirect effect represents the pathway mediated by female obesity. Confidence intervals were estimated using bootstrapping (5000 resamples).

## Data Availability

The data used in this study are publicly available from international databases. Childhood and female obesity data were obtained from the World Health Organization Global Health Observatory (https://www.who.int/data/gho, accessed on 26 March 2026). Food system indicators, including sugar and vegetable oil availability and pesticide use, were obtained from FAOSTAT (https://www.fao.org/faostat/, accessed on 26 March 2026). Socioeconomic indicators, including GDP per capita, urbanization, and female educational attainment, were obtained from the World Bank Open Data platform (https://data.worldbank.org/, accessed on 26 March 2026). The compiled dataset used for the analyses is available from the corresponding author upon reasonable request.

## Supplementary Materials

The following supporting information can be downloaded at https://www.mdpi.com/article/10.3390/nu18091435/s1, Table S1: Countries included in the study sample, grouped according to World Bank income classification.
