# Supplementary material for: Industrial Food Systems as Environmental Drivers of Women’s Health and Childhood Obesity: A Cross-National Ecological Study
Source: Nutrients. 2026 Apr 30;18(9):1435. doi: 10.3390/nu18091435 (PMC13164980; doi:10.3390/nu18091435)
Supplement: Supplementary file 1 [file nutrients-18-01435-s001.zip › nutrients-4254276-supplementary.pdf]

Supplementary Table S1. Countries included in the study sample, grouped according to World Bank income classification.

Income category	n	Countries included
Low-income countries	3	Ethiopia, Haiti, Uganda
Lower-middle-income countries	8	Egypt, India, Indonesia, Morocco, Nepal, Nigeria, Philippines, Viet Nam
Upper-middle-income countries	16	Argentina, Brazil, China, Colombia, Costa Rica, Dominican Republic, Ecuador, Iran (Islamic Republic of), Kazakhstan, Malaysia, Mexico, Paraguay, Peru, South Africa, Thailand, Türkiye
High-income countries	19	Austria, Belgium, Canada, Chile, Germany, Ireland, Italy, Japan, Netherlands (Kingdom of the), Poland, Portugal, Republic of Korea, Romania, Russian Federation, Spain, Sweden, United Kingdom of Great Britain and Northern Ireland, United States of America, Uruguay
**TOTAL**	**46**	

Note: Income categories were assigned according to World Bank classifications used during the study period.
